# Gavi HPV Programs: Application to Implementation

**DOI:** 10.3390/vaccines3020408

**Published:** 2015-05-20

**Authors:** Celina M. Hanson, Linda Eckert, Paul Bloem, Tania Cernuschi

**Affiliations:** 1 Gavi The Vaccine Alliance Secretariat, 2 Chemin des Mines, Geneva 10 CH-1211, Switzerland; E-Mail: cmh558@nyu.edu; 2 Department of Obstetrics and Gynecology, University of Washington, Box 359865, 325 9th Avenue, Seattle, WA 98104, USA; 3 World Health Organization, Department of Immunization, Vaccines and Biologicals, 20 Avenue Appia, Geneva 27 CH-1211, Switzerland; E-Mails: bloemp@who.int (P.B.); cernuschit@who.int (T.C.)

**Keywords:** HPV, vaccination, cervical cancer

## Abstract

Developing countries disproportionately suffer from the burden of cervical cancer yet lack the resources to establish systematic screening programs that have resulted in significant reductions in morbidity and mortality in developed countries. Human Papillomavirus (HPV) vaccination provides an opportunity for primary prevention of cervical cancer in low-resource settings through vaccine provision by Gavi The Vaccine Alliance. In addition to the traditional national introduction, countries can apply for a demonstration program to help them make informed decisions for subsequent national introduction. This article summarizes information from approved Gavi HPV demonstration program proposals and preliminary implementation findings. After two rounds of applications, 23 countries have been approved targeting approximately 400,000 girls for vaccination. All countries are proposing primarily school-based strategies with mixed strategies to locate and vaccinate girls not enrolled in school. Experiences to date include: Reaching marginalized girls has been challenging; Strong coordination with the education sector is key and overall acceptance has been high. Initial coverage reports are encouraging but will have to be confirmed in population based coverage surveys that will take place later this year. Experiences from these countries are consistent with existing literature describing other HPV vaccine pilots in low-income settings.

## 1. Introduction

Dealing with cancer is a source of anxiety and suffering for people and their families in any country. Yet, dealing with the disease in low-income countries, where morbidity and mortality is highest due to resources and health system constraints has additional challenges. This is especially evident with cervical cancer, which has often been coined the “disease of disparity” [1]. In 2012, there were an estimated 528,000 new cases of cervical cancer worldwide with nine out of 10 cervical cancer deaths occurring in less developed countries [2].

These patterns of disease incidence and mortality are largely a result of the scope and quality of prevention and care efforts. A range of cervical cancer screening methods are available to diagnose cervical cancer: Cytology, visual inspection and the Human Papillomavirus (HPV) DNA test. Indeed, screening methods have decreased cervical cancer incidence and mortality in high resource settings; however, cervical cancer screening has not impacted cervical cancer mortality in many low-income countries mainly due to the lack of availability of screening and treatment for pre-invasive disease [3].

Two WHO prequalified HPV vaccines are available that protect against the main genotypes causing cervical cancer: Cervarix^®^ and Gardasil^®^. Cervarix^®^ is a bivalent vaccine that protects against, HPV types 16 and 18. Gardasil^®^ is a quadrivalent vaccine that provides additional protection against HPV types 6 and 11, which can cause genital warts, low-grade cervical cell change and respiratory papillomatosis [4,5,6]. The majority of cervical cancers are caused by types 16 and 18 of the human papillomavirus (HPV) [7]. These have also been shown to cause oropharyngeal and anogenital cancers [8,9]. Studies have shown that both vaccines are safe, immunogenic and efficacious and may provide cross-protection against other HPV genotypes [10]. Both vaccines are recommended for girls aged 9 to 13 year olds by the World Health Organisation (WHO) [11].

These vaccines were first introduced in high-income countries in 2006 [12], yet their use has been very limited in low-income countries due to the high vaccine price and challenging delivery logistics. Gavi The Vaccine Alliance, a global public private partnership aimed at bridging the gap between introductions of new vaccines in high- and low-income countries, worked with vaccine manufacturers to markedly reduced the price of both vaccines to less than US $5 per dose [13] in Gavi-eligible countries, where the disease burden is highest [14,15,16]. In November 2011, the Gavi Board opened a funding window to provide support to countries interested in introducing HPV vaccination.

## 2. Gavi HPV Vaccine Programs

**Figure 1:**
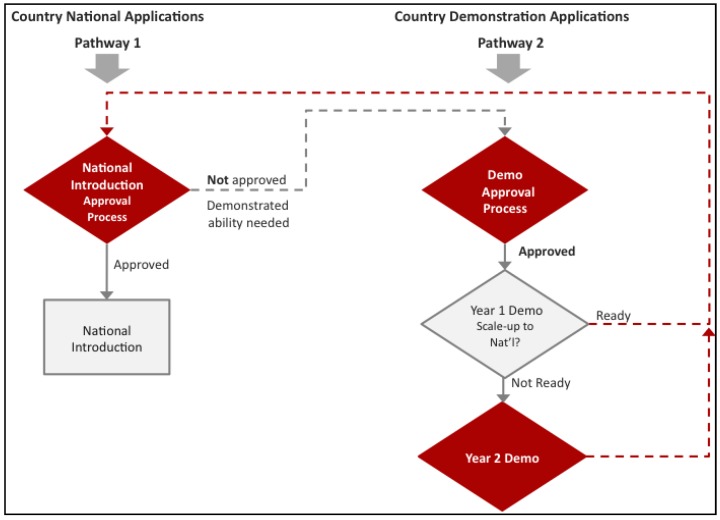
Gavi has developed two pathways for Human Papillomavirus (HPV) vaccine support. **Pathway 1** outlines traditional Gavi support for national introduction. This pathway requires countries to not only meet the Gross National Income per capita (GNIpc) of US $1580 and a Diphtheria-Tetanus-Pertussis third dose (DTP3) coverage of at least 70%, but to have also demonstrated the ability to deliver a multi-dose vaccine to at least 50% of a target population of 9–13 year old girls in an average district size. For countries that do not have this experience, Gavi devised a second pathway. **Pathway 2** gives countries the opportunity to gain experience vaccinating this cohort before they decide on whether they would like to introduce HPV vaccine nationally.

**Figure 2:**
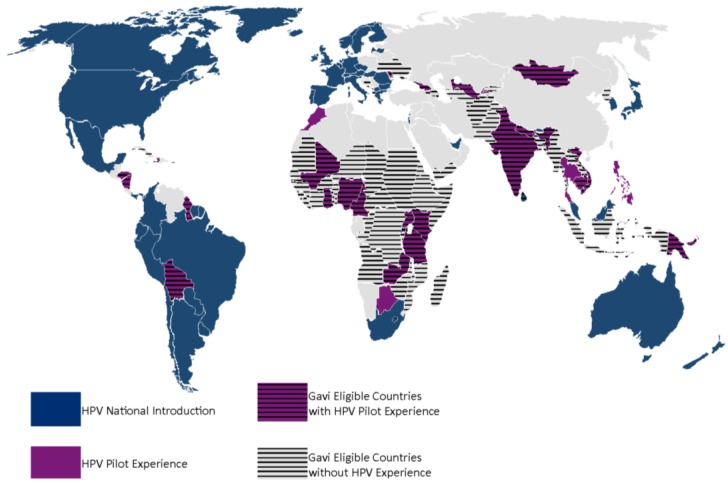
Global map showing HPV vaccination experience in Gavi eligible and non-eligible countries. As of 1 January 2015, only three Gavi eligible countries have introduced HPV nationally, Bhutan, Lesotho and Rwanda. The majority of Gavi eligible countries have not had experience with HPV vaccination. Few Gavi eligible countries have had pilots previous to the development of the Gavi HPV demo program and without Gavi support.

Normally, for other routine vaccines, Gavi funds countries for national introduction. For national HPV vaccine support, Gavi not only requires countries to meet the Gross National Income per capita (GNIpc) of US $1580 and a Diphtheria-Tetanus-Pertussis third dose (DTP3) coverage of at least 70% as for all other vaccines, but to have also demonstrated the ability to deliver a multi-dose vaccine to at least 50% of a target population of 9–13 year old girls in an average district size [17]. In the case of HPV vaccines, Gavi through its partners, the WHO, BMGF, PATH, UNICEF, and UNFPA, designed an additional support pathway in the form of a demonstration project (Figure 1). This pathway would give countries the opportunity to gain experience with the HPV vaccine, since many Gavi eligible countries have not had experience with HPV vaccinations (Figure 2) or other multi-dose vaccinations in this age group. Also, immediate national introduction without prior experience presents distinct challenges in delivering vaccines to young adolescents, a cohort not normally serviced by routine immunization nor by many other health interventions [18,19]. Additionally, the vaccine protects against a sexually transmitted virus about which many communities have little knowledge especially it’s link to cervical cancer [20]. Finally, experiences from many countries in diverse geographic locations have demonstrated that the initial HPV vaccination efforts provide excellent opportunities to learn effective communication strategies and improve delivery methods thereby increasing vaccine coverage [21,22]. All of these “lessons learned” then inform national policymaking, and can be leveraged for national scale-up. By contrast, in some countries that did not conduct pilots; low initial vaccine acceptability could be linked to implementation challenges that may have been prevented [23,24].

The Gavi HPV demonstration program encourages countries to take part in a two-year project to “learn by doing”. To be eligible for the demonstration program, a country must have at least 70% Diphtheria-Tetanus-Pertussis third dose (DTP3) coverage at the national level, an indicator chosen as a proxy for the strength of their immunization systems. The demonstration program requires countries to implement vaccination and to evaluate its coverage, feasibility, acceptability and cost and then take an informed decision about national introduction. The program also requires countries to review options to integrate HPV vaccination with provision of additional adolescent health services to both girls and boys with the goal of enhancing efficiency and sustainability of these interventions. Finally, the program encourages development of a comprehensive national cervical cancer prevention and control strategy integrating HPV vaccination as primary prevention.

The costs of HPV vaccination vary widely depending on many factors such as existing vaccine infrastructure, population density, and delivery strategy [25]. Estimated introduction costs for national scale-up of HPV vaccinations in low income countries range from US $3.13 to US $5.15 per fully immunized girl and US $4.23 to US $5.81 for operational costs per fully immunized girl [25]. For its demonstration program, Gavi covers the entire cost of vaccine supply and procurement until port of entry. In addition to vaccine supply, Gavi provides a cash grant to countries to support approximately 80% of start-up and operational costs of vaccine introduction in the districts of the demonstration program. Countries choose how to make use of the grant and are asked to take ownership of the HPV demo program by covering the remaining costs. For the national introduction, Gavi requires countries to co-finance the cost of the vaccine based on their national wealth, measured in Gross National Income (GNI) per capita, and provides a Vaccine Introduction Grant (VIG) aimed at covering 80% of the start-up cost of vaccine introduction (countries cover program recurrent costs) [26].

Gavi also provides funding to partner organizations to provide technical assistance to countries for application preparation and for implementation and evaluation support. These funds will allow countries to secure relevant technical support. Countries may also secure additional limited support from other sources for project activities.

## 3. Applications

### 3.1. Overview

After two opportunities to apply, in the summer of 2012 and of 2013, 20 countries have been approved for demonstration programs and three countries, Rwanda, Uganda and Uzbekistan, were approved for national introduction support. Table 1 summarizes the Gavi HPV demonstration application components [27]. For demonstration programs, most countries are targeting two districts with an average target population of 10,000 girls per year. Districts have been chosen based on representativeness in terms of socio-economic and ethnic mix; urban-rural mix; current strength in cold chain capacity; and functioning vaccine infrastructure evidenced by DTP3 coverage greater than 70%.

The majority (67% or *n* = 16) of countries who have applied for support stated a primary preference for the quadrivalent vaccine. This preference held across all regions, with the exception of South and Central Africa. Reasons stated for choosing the quadrivalent formulation were the extra protection against types 6 and 11, prior experience and the vaccine being already registered in the country. Countries chose the bivalent vaccine due to its 2-dose vial presentation making it less demanding for cold chain storage, and the assumption of low prevalence of HPV types 6 and 11 in country.

### 3.2. Target and Delivery Strategy

As per program requirements, countries are devising strategies to reach adolescent girls including marginalized or hard-to-reach girls. Therefore, all countries have proposed more than one or a mixed approach to reach adolescent girls. Most applications proposed a school-based strategy as their primary strategy with a preference for vaccinating 10 years olds. The target age is proposed to capture girls before sexually active and while they remain in school.

The primary strategy is intended to reach the majority of girls; however, in line with Gavi’s policy to immunize every child, countries are required to propose immunization strategies, which reach marginalized or hard-to-reach girls. Countries propose several strategies and methods to locate these hard-to-reach girls, which include the use of census or registries, enumeration of girls by community health workers, and mapping health facility areas and defaulter tracing, in which health care workers go into communities to follow up with girls who did not receive their consecutive doses.

Following the process of locating these hard-to-reach girls, countries are proposing a mix of fixed and outreach strategies to vaccinate these girls. Fixed strategies involve using a designated place, such as schools, health facilities or a village square, as a hub in which girls would be directed to go for vaccinations. In outreach strategies, vaccinators travel to outlying communities.

**Table 1:** Summary of Gavi Approved HPV Demonstration Program Proposals [27].

Country	Started Implementation *	Previous HPV Pilot	Est. Target Population	Vaccine Preference	Selected District(s)	Target Grade	Target Age	Primary Intro Strategy	Marginalized or Out of School Strategy
**Benin**	No	No	13,377	Bivalent	Commune V du District de Bamako and Fana	N/A	9 yrs	School, age based	Health facility and Outreach
**Burundi**	No	No	21,812	Bivalent	Ngozi and Rumonge	Primary 3	10 yrs	School, grade based	Health facility
**Cameroun**	Yes	Yes	31,876	Quadrivalent	Edea and Foumban	Primary 6	10 yrs	School, grade based	Health facility and Outreach
**Côte d'Ivoire**	No	No	27,121	Quadrivalent	Abengourou and Korhogo	N/A	10 yrs	School, age based	Health facility
**Gambia**	Yes	No	12,213	Quadrivalent	Brikama	Primary 3	9 yrs	School, grade based	Health facility
**Ghana**	Yes	Yes	12,432	Quadrivalent	Ningo-Prampram, Shai-Osudoku, Tamale Metro, and Sangregu	Primary 6	11 yrs	School, grade based	Health facility
**Kenya**	Yes	Yes	17,242	Quadrivalent	Kitui	Primary 4	10 yrs	School, grade based	Health facility
**Lao PDR**	Yes	No	28,224	Quadrivalent	Vientiane Municipality and Vientiane Province	Primary 5	10 yrs	School, grade based	Refer to school and Outreach
**Liberia**	No	No	28,735	Quadrivalent	Bong and Nimba	N/A	10 yrs	School, age based	Health facility and Outreach
**Madagascar**	Yes	No	15,000	Bivalent	Toamasina 1 and Soavinandriana	Primary 5	10 yrs	School, grade based	Health facility and Outreach
**Malawi**	Yes	No	21,862	Quadrivalent	Zomba and Rumphi	Primary 4	10 yrs	School, grade based	Refer to school, Health facility and Outreach
**Mali**	Yes	Yes	25,812	Quadrivalent	Commune V du District de Bamako and Fana	N/A	10 yrs	School, age based	Health facility and Outreach
**Mozambique**	Yes	No	5659	Bivalent	Manhica	N/A	10 yrs	School, age based	Health facility and Outreach
**Niger**	Yes	No	39,099	Quadrivalent	Niamey 2 and Madarounfa	N/A	11 yrs	Campaign	Campaign
**Senegal**	Yes	No	9274	Quadrivalent	Mekhe and West Dakar	N/A	9 yrs	School, age based	Health facility and Outreach
**Sierra Leone**	Yes	No	20,646	Bivalent	Bo	N/A	9 yrs	School, age based	Health facility and Outreach
**Solomon Islands**	Yes	No	10,000	Quadrivalent	Isabel and Honiara Town Council	N/A	9–12	School, age based	Health facility and Outreach
**Tanzania**	Yes	Yes	29,568	Quadrivalent	Moshi urban, Moshi rural, Hai and Siha, Rombo (4 districts)	Primary 4	9 yrs	School, grade based	Refer to school and Health facility
**Togo**	No	No	25,911	Bivalent	Golfe and Tchamba	N/A	10 yrs	School, age based	Health facility
**Zimbabwe**	Yes	No	8882	Bivalent	Beitbridge and Marondera	Primary 5	10 yrs	School, grade based	Health facility and Outreach

* Implemented between April 2013 to January 2015.

### 3.3. Integrating HPV Vaccination with Adolescent Health

Gavi advocates for adolescent health stakeholders to be involved in the demonstration program from the onset. The demonstration program provides an opportunity to consider the delivery of other health interventions concurrent with HPV vaccine administration. This may both provide an opportunity to increase efficiency and sustainability of HPV vaccination and improve adolescents’ access to other health interventions [28,29]. To facilitate this partnering of HPV vaccine delivery with other health interventions, Gavi requires countries to carry out an assessment of the feasibility, in the country’s context, to integrate an adolescent health intervention with HPV vaccinations.

Within Gavi, a requirement to consider linkages beyond the vaccination itself is new and only found in the HPV vaccine demo program. Given that this is a new requirement, few countries have experience with these linkages. Countries have responded to this idea with enthusiasm, proposing several interventions for adolescent health integration in their applications to Gavi (Box 1).

Box 1 **Potential Adolescent Health Services or Interventions [27].**

- Sexuality education
- Personal and menstrual hygiene education
- Prevention of tobacco and other drug use
- Gender based violence prevention
- Education on adolescent rights
- Soil transmitted helminthes and schistosomiasis treatment
- Nutrition supplementation and education
- Insecticide treated bed nets for malaria prevention
- Tetanus vaccinations

### 3.4. National Cervical Cancer Control Plans

A national cervical cancer control plan links primary and secondary strategies to prevent cervical cancer into a comprehensive strategy for cervical cancer prevention and control. In contrast to integrating adolescent health interventions with vaccinations, this Gavi requirement to develop or strengthen an integrated approach to cervical cancer prevention and control was more familiar to applying countries. In fact, twelve of the 23 country applicants currently have national cancer control plans that include cervical cancer. Cervical cancer screening features in many of these plans and a few countries mention integration with cervical cancer education and the possibility of a mother-daughter approach or simultaneous screening for mothers and vaccinations for daughters. The countries that have no national plans provided detailed strategies to develop cervical cancer prevention and control plans.

## 4. Implementation

Experiences to date of the Gavi HPV support programs are based on preliminary informal results compiled from country feedback gathered in bi-weekly country update calls, workshops and written communication. More robust coverage and costing evaluations are required by Gavi and will be conducted by countries by the end of the first year of vaccination. By January 2015, fifteen (Table 1) countries have launched HPV vaccination with Gavi demonstration program support. Only one country has launched their national HPV vaccine introductions with Gavi support in 2014, however, no data is currently available from this program. The other two countries plan on introducing national HPV vaccinations at the end of 2015 and early 2016.

In early 2014, SAGE made recommendation for a 2-dose schedule for both available vaccines for girls 9–13 years of age [30]. Gavi adopted the SAGE recommendations and allowed approved countries the choice to start demonstrations on a 3-dose schedule or to switch to a 2-dose schedule. Ghana, Kenya, Lao, Madagascar, Malawi, Niger, Sierra Leone and Zimbabwe implemented a 3-dose schedule. The remaining five countries implemented with a 2-dose schedule.

To date, the acceptance and uptake of the HPV vaccine has been high, and in some cases exceeding other low-income countries’ early experience [31]. Two countries reported refusals in some private or faith-based schools. These countries mentioned that spending more time on social mobilization and advocacy activities with authorities, including religious authorities, was necessary in garnering cooperation from these schools. Negative rumors about the vaccine have also been reported in six countries. Countries strengthened communication activities to prevent or dispel rumors and myths and have found that this is an important component of well performing HPV vaccine programs.

In the first stage of the demonstration programs, seven countries have reported challenges in identifying effective strategies for reaching marginalized or out-of-school girls. Included in this marginalized group are children living on the streets without parents or guardians and migrating populations, for which appropriate delivery strategies were not considered in advance.

## 5. Discussion

It is too early to conclude whether the Gavi HPV demonstrations are meeting their objectives and preliminary findings from countries correspond with current published data from India, Peru, Uganda and Vietnam [31,32,33]. Once the data of HPV coverage, feasibility, acceptability and cost surveys by countries are available, and this experience informs strategic changes to reach program objectives, the success of demonstration programs will become clearer. Armed with the data and learning from these demonstration projects, countries may make an informed choice to move ahead with national introduction, apply for another demo or otherwise decide not to introduce HPV vaccination nationally. In the interim, much can be learnt from the applications and current country experiences.

The micro planning involved in these vaccination programs encompass a myriad of technical components necessary to orchestrate vaccination in this target population. Much forethought is required to meet program objectives in training, sensitization, technical assistance, evaluation, and consideration of the finer details at the district level like social mobilization, delivery, and alignment with the school calendar. In addition, the HPV programs are competing with school priorities like exams and holidays and other national priorities. In some countries, disease outbreaks have affected or temporarily halted vaccine implementation. Therefore, the emphasis the program places on micro planning in accordance with school calendars may assist in the delivery of vaccine with timely demonstration project initiation.

During the implementation phase, countries are beginning to learn more about the challenges in identifying and reaching marginalized, out of school adolescent girls. Determining the location of these girls and overcoming the obstacles they face in accessing basic health services is one of the aims of the program. With limited experience vaccinating and locating this age group of girls, it is not surprising that countries have encountered difficulty accurately allocating resources to target this population. We will gain more insight into how countries address these marginalized girls as countries progress towards the second year of the program. Countries have a real opportunity to learn how to implement a national vaccination program by aiming to maximize coverage, rather than the easiest methods of capturing just the majority. In fact, thus far, some countries have made efforts to vaccinate every girl listed on registries; however, this has led to higher than expected costs. The challenge for countries moving into their second year of the demo project is to critically evaluate current strategies and adapt them further to arrive at sustainable approaches that may effectively inform national introduction.

To facilitate the learning process, Gavi and partners have been linking program countries in a peer learning approach. Countries benefit from a timely and critical assessment of their technical support by working with Gavi and its technical partners to address these needs. In turn, Gavi is strengthening and better coordinating technical support by improving communication with countries to better explain all the nuances of this program. Evaluating these needs in a compressed time frame for this HPV program has not been previously required for other vaccine programs, and hence is new for countries and Gavi alike.

In a few countries, national or expanded program for immunization (EPI) are not leading the implementation of the demonstration projects. They are being led by cancer or women’s and adolescent health departments that have not traditionally been involved in vaccinations. Forging these new connections have made coordination of vaccine delivery between partners challenging. The willingness of other departments besides EPI to lead these programs demonstrates the broad stakeholder involvement associated with HPV not present in many other vaccination programs. However, the national introduction HPV vaccine delivery will eventually fall on EPI programs, which is another consideration for countries planning to introduce nationally.

Overall, Gavi’s HPV vaccination programs have led to an increase in awareness of vaccine preventable cancers and may also lead to increased attention to the health of adolescents, a population that is often ignored and underfunded by governments [19,34]. Gavi’s objective to ensure HPV vaccination is a platform for integrating adolescent health interventions puts the Alliance in a unique position to be a catalyst for countries to explore the feasibility of this approach. Similarly, the demo program strengthens national comprehensive cervical cancer prevention and control plans in countries and contributes toward the Global Action plan on NCDs national agendas.

## 5. Conclusions

The HPV vaccine demonstration programs are a learning opportunity for countries applying for support as well as for Gavi. The demonstration program has many components and hence requires more planning, making it different from existing vaccine support programs. The unique feature of HPV vaccination as a component of an integrated approach holds promise for application with other vaccines. Gavi and its technical partners have offered considerable support and monitoring of progress in the many technical aspects and evaluations. This has required close communication between partners in countries and at international level. Moving forward, as more country level data and experiences become available, countries will need to fine-tune their approaches and ensure they continue to “learn by doing”. Through the demonstration program, Gavi provides countries with a unique opportunity for learning in the country context.
